# Informed consent in dementia research: how Public Involvement can contribute to addressing “old” and “new” challenges

**DOI:** 10.3389/frdem.2025.1536762

**Published:** 2025-02-04

**Authors:** Ana Diaz, Cindy Birck, Angela Bradshaw, Jean Georges, Daphne Lamirel, Soraya Moradi-Bachiller, Dianne Gove

**Affiliations:** Alzheimer Europe, Senningerberg, Luxembourg

**Keywords:** Public Involvement, informed consent, research, dementia, lived experience

## Abstract

Informed consent is a critical ethical requirement in research, ensuring the protection of participants' rights and promoting their wellbeing and autonomy. In dementia research, this process becomes particularly complex due to cognitive impairments and fluctuating capacity. While substantial work has been done to address these challenges, much of the literature on informed consent in dementia research has been shaped by the perspectives of researchers and healthcare professionals, with less focus on those with lived experience. This paper provides an overview of the perspectives of people with dementia and their carers resulting from Public Involvement activities organized by Alzheimer Europe. It builds on Alzheimer Europe's previous work with the European Working Group of People with Dementia and draws on discussions held during a face-to-face meeting about Participant Informed Consent forms and processes used in two specific European projects. We highlight views and key concerns raised by people with lived experience regarding the informed consent process, including barriers and facilitators. In addition to ensuring understandability, the discussions emphasized the importance of promoting respect and autonomy, ensuring that the values and interests of people with lived experience remain central throughout the research process. This paper contributes to the ongoing dialogue on improving informed consent practices in dementia research, highlighting the need for continuous involvement and the inclusion of people with lived experience in shaping consent practices to address both old and emerging challenges (i.e., new types of research such as artificial intelligence and data sharing/re-use) in dementia research.

## 1 Introduction

Informed consent is one of the most fundamental conditions for the ethical conduct of research, ensuring that participants' rights, wellbeing, and autonomy are promoted. It is not only an ethical necessity but, in some instances, also a medico-legal obligation to prevent exploitation and provide information about potential harm (i.e., linked to preventing abuse, deception, illegal experimentation, and the charge of physical assault). Informed consent must be obtained before the participant enters the research study and should provide full information so that potential participants understand what the research is, what they are consenting to and the voluntary nature of their participation and possible withdrawal. The process typically involves three stages: (1) disclosing the information needed to make an informed decision about participation, (2) a discussion to address any questions or concerns that may arise, and (3) obtaining formal consent from the person (or a proxy), voluntarily confirming their willingness to participate.

Due to the nature of dementia and associated symptoms and impairments, informed consent for dementia research can present significant challenges. Issues surrounding capacity are a common concern for dementia researchers and have been an important focus of research work and legislation over past decades. Over the years, a very important and relevant amount of work has been conducted to address the complex nature of consent for people with dementia, understand the practical and ethical challenges, and provide guidelines on how to assess and address capacity during this process (e.g., Hubbard et al., 2003; Hellström et al., 2007; Dewing, 2008; Nuffield Council on Bioethics, 2009; Beattie, 2009; Howe, 2012; Alzheimer Europe, 2019; O'Connor et al., 2022; Tauzer et al., 2023; Pyer and Ward, 2023).

Alzheimer Europe (2019, 2020, 2023) and other organizations have highlighted that decision-making and capacity should not be considered as an “all or nothing” or “one-off” event but as an ongoing process, taking into consideration that decision-making is task-specific i.e., related to performing “a particular decision-making task at a particular time and under specified conditions” (Buchanan and Brock, 1990, p. 18) and that capacity can fluctuate. Such considerations are therefore central to informed consent and the inherent imperative to promote autonomy. Recent studies looking at the views, perspectives and concerns of people with dementia in relation to consent have shown that people with dementia and carers describe the consent process as a journey. This work also highlights the value and importance of taking a flexible approach to consent (Pyer and Ward, 2023). As suggested by Hellström et al. (2007), the question therefore should no longer be whether people with dementia should be included in research, but rather how we can best achieve this.

The progressive nature of dementia calls for continuous engagement with research participants, including regular monitoring of their capacity and willingness to continue participating, as well as considering different possible levels of support for decision making when and if needed. Concepts such as “adapted consent” (Alzheimer Europe, 2019), “person-centered/process consent” (Dewing, 2008) and “supported decision making” (Alzheimer Europe, 2019; Griffiths et al., 2022) have all been developed as part of this work and efforts made by researchers to address this complex issue.

There are also broader considerations related to capacity and consent. People with dementia should have an equal right to accept risks in the context of research and in so doing, to contribute toward scientific progress. However, it is important to protect potential participants from therapeutic misconceptions and from exaggerated claims about the benefits of the research. Altruism is a frequently cited motive for taking part in research but having a terminal condition puts people in a vulnerable position with regard to accepting risk. The informed consent process should help ensure that any unrealistic expectations, fears or misguided beliefs about the nature of research do not interfere with making truly informed decisions that are in keeping with people's values and personal interests.

While there is quite a lot of research and literature on informed consent in dementia research, most of this work has been shaped by the perspectives of researchers and healthcare professionals. In this article, we would like to contribute to these discussions surrounding informed consent in dementia research by summarizing the views and concerns expressed by people with lived experience in the context of Public Involvement (PI) activities, conducted by Alzheimer Europe (AE), in different European projects.

In addition, the field of dementia research is rapidly evolving with the emergence of new types of research and study designs (e.g., involving people at risk of dementia with no symptoms, artificial intelligence, and data sharing/re-use). These changes have exacerbated some of the existing challenges in obtaining informed consent and introduced new concerns. In this article, we therefore explore how PI work can contribute toward the understanding and conceptualization of consent in the light of existing and new challenges.

## 2 Approach

Although PI is not the same thing as qualitative research, a qualitative approach/methods can be used. PI is about involving people with dementia in the research process, but not as participants. It is about creating a partnership between researchers and members of the public, whereby all contribute collaboratively in varying degrees toward the research process or the research output. AE has promoted PI in dementia research for over a decade (Gove et al., 2018). Examples of PI activities conducted in the context of European-funded research projects include, among other activities, the review of research protocols and participant-facing materials, participation in the process of selecting devices to be used in the study, discussions related to recruitment and retention strategies planned for the study, as well as discussions related to ethical issues linked to the study or future use of the project-related outcomes (Owens et al., 2020; Diaz et al., 2021; Brem et al., 2023; Muurling et al., 2023).

Through the active involvement of members of the European Working Group of People with Dementia (EWGPWD, https://www.alzheimer-europe.org/about-us/european-working-group-people-dementia) and various project-specific Advisory Boards, AE has facilitated meaningful involvement of people with dementia and carers in European research projects.

- The EWGPWD is composed of 14 people with dementia from different European countries and with different types of dementia. Members are nominated by a national Alzheimer Association for a term of office of 3 years. The group meets regularly including face-to-face and online meetings. Members can be supported for travel and at meetings by a person of their choice, usually a relative, friend or member of an Alzheimer organization. In this article, we refer to the person providing support to the person with dementia as carer/supporter.
- The Advisory Boards are composed of people with Mild Cognitive Impairment (MCI) due to Alzheimer's disease (AD), people with dementia and carers, and are set up to provide feedback and advice to a specific project. The number of members of the Advisory Board can vary, typically ranging from 7 to 15 members.

This article draws on the discussions at a face-to-face meeting held on 15 and 16 November 2023 in Luxembourg, on the topic of informed consent in dementia research, in the context of two ongoing European research projects: EPND and ADIS. ADIS is a European Union Joint Programme—Neurodegenerative Disease Research (JPND)-funded project aiming at characterizing the role of peripheral blood cytotoxic lymphocytes as potential biomarkers for the early prediction of AD, and to investigate the influence of sleep disturbances on these biomarkers. EPND is an Innovative Medicines Initiative (IMI) project that is developing a platform for researchers to share data and biosamples from neurodegenerative disease studies so that these can be (re)used for further research. AE has led PI activities in both projects addressing in this work a broad number of topics such as, for example, ethical challenges linked to the early diagnosis of Alzheimer's disease in ADIS and to data sharing/re-use in EPND.

The meetings in Luxembourg were facilitated by AE staff, and involved a total of 29 people including people with dementia (members of the EWGPWD), people with MCI due to AD and the supporters/carers of the people with dementia/MCI due to AD.

This paper summarizes some of the discussions that took place during this meeting, highlighting how informed consent was perceived by these people with lived experience and what they felt were the more relevant current and future challenges related to this topic. The discussions were based on issues linked to the Informed Consent forms used in the ADIS and EPND projects. In the case of EPND, this referred to consent to secondary use of data and data sharing. In addition, there was a broader question about how they perceived informed consent and the main concerns and issues that need to be addressed, including barriers and facilitators for involving people with cognitive problems/dementia in this process. The paper also builds on AE's previous PI work in the context of several research projects (https://www.alzheimer-europe.org/our-work/current-work) with members of the EWGPWD over the years for which the topic of consent, whilst not the key topic, was also discussed and therefore reflects an ongoing dialogue on the topic (see for example Muurling et al., 2023 in the context of the Innovative Medicines Initiative (IMI)-funded project RADAR-AD, https://www.radar-ad.org/).

In this paper, we use the term “people affected by dementia” to refer collectively to the views and concerns of people with dementia, MCI due to AD and carers/supporters involved in this work.

## 3 Key issues related to informed consent raised by people affected by dementia

Discussions with people affected by dementia emphasize the great relevance of the topics of research and informed consent. Access to research can be hindered not only by practical factors (e.g., lack of research opportunities) but also, by misconceptions about dementia and about the capacity, abilities, and willingness of people with dementia to contribute to research. Unfortunately, dementia is still often portrayed and perceived by many people as the moderate and especially the late stages of the disease. An important message that emerged from the discussions was that, without denying or neglecting the challenges that people with dementia may experience, the focus should be on how to promote and enable participation in research for those who are interested and willing to participate. Enablers can include, for example, advance directives where the person could indicate in advance their wishes about research participation in the future when the condition progresses, but also reflections on how to promote and support autonomy and meaningful decision-making processes. The following sections summarize four important topics related to informed consent raised by people affected by dementia:

1. Broadening the understanding of informed consent.
2. Supporting the “informed” part of the informed consent process.
3. Beyond the provision of information: Promoting respect, recognition and wellbeing.
4. New research approaches will affect the consent process in dementia.

### 3.1 Broadening the understanding of informed consent

Consent has usually been conceptualized as a process starting just before a participant enters a research study, focused solely on that particular study. However, other broader elements, not specifically related to the study, such as awareness of the general public about research, opportunities to access research and “normalizing research” can all play an important role and should also be considered when planning informed consent materials and procedures. Many members of the public, including people affected by dementia, have limited awareness of research, lack understanding about its value, and sometimes, have misconceptions or fears about research and research participation. Being “research-aware” and understanding what it entails and its value, could influence trust and make people more open and better prepared to make informed decisions about participating in research. In addition to this, people affected by dementia should be involved in developing research materials (e.g., informed consent forms). This could help “normalize” research, thereby making it more inclusive and appropriate for people with dementia.

“*I'm part of a Dementia Research Advisory Team, so if researchers want to do a new piece of research, they talk to us. This normalizes research.”*

“*Often doctors don't know much about research. It needs to be normalized, and Public Involvement needs to happen early. We can help develop consent forms.”*

### 3.2 Supporting the “informed” part of the informed consent process

An essential aspect of consent is that it is “truly” informed. The specific needs and preferences of people affected by dementia should be considered and the process should be flexible and adapted to such needs and wishes.

“*Informed” is the crucial part of the term informed consent.”*

“*Everyone has to be on an equal footing.”*

“*There is no one size fits all. The process (for informed consent) needs to take this into account and be flexible and adaptable.”*

In line with this, a key priority for people affected by dementia relates to how to facilitate and support comprehension and understanding in a respectful and meaningful manner. Aspects that can facilitate understanding and accessibility include the terminology used and the length and layout of the document, but also more subtle elements such as the complexity of the content and the tone/style used.

#### 3.2.1 Language and jargon

All participant-facing documents should be clearly worded, in lay language, avoiding technical terms and jargon, and phrased in a way that does not assume any prior knowledge. At the same time, it is also important not to assume that everyone lacks knowledge, as some people may be familiar with some of the technical or medical terms used. Glossaries and lay summaries were suggested as possible ways to support potential participants in the consent process whilst recognizing their different abilities, skills and needs.

“*If instead of CSF, “lumbar puncture” was in there, I would have caught this.”*

“*I think the glossary is a good idea and it helps with having a balance because some people might already know some of the more technical terms.”*

#### 3.2.2 Length

Another important issue is that informed consent forms are often excessively long and this may put people off reading the whole text or make it difficult to read. This is particularly relevant for people with cognitive problems but it can also be an issue for carers and other potential participants who do not have any cognitive issues. The issue of length was also related to the amount and type of detail included. It was acknowledged that researchers may need to include certain information or details as these may be required by Research Ethics Committees, but, at the same time, there was a concern that the information that is emphasized may not necessarily correspond with participants' needs, priorities or what is meaningful and relevant for them. Finally, it is important to consider the complexity of the topics addressed as certain topics, such as risk, privacy, artificial intelligence, or data protection, can be very technical, and some people may find it difficult to make sense of them or understand their potential implications.

“*For me, just in general, the whole thing's too long. If I got that as a carer, I just wouldn't. Honestly, I wouldn't read it all, because I just wouldn't have the time to do it. (…) I just think there's a lot in the beginning that I probably want to just quickly flash through, and then I would have signed.”*

“*The medical part is ok… but you are more interested in what concerns you, what affects you directly.”*

“*Regarding information about how people's data is stored, I don't know if people will have any know-how in technical issues and data security.”*

#### 3.2.3 Tone of the document

The tone of the document (e.g., friendly, formal or academic) and the layout were perceived to be as important as the issues linked to terminology and length. An academic, medical or formal style of writing (even if jargon is not used) can make the text much more complex, difficult to read and potentially daunting, and make people more uncomfortable or ill at ease, whereas a more informal writing style can help the person to read faster and more confidently.

“*The way the document (the ICF) is written is very medical, ‘legalistic.' It would be easier to read if it was written in a friendlier and warmer manner.”*

#### 3.2.4 Presentation: layout and visuals

The layout of the text is also important. Highlighting the most important sections, breaking down the document in smaller chunks of information, using visuals and colors to help the person understand when one topic ends and a new one begins, and using different strategies or methods for providing information, were all described as ways of facilitating understanding. The use of visuals can be particularly helpful when discussing the topic of risk in different contexts. For example, visuals which use a traffic-light inspired approach using the colors red (high risk), amber (moderate risk), and green (lower risk) to visually indicate different levels of risk could be helpful to explain risk to participants in a simple manner. This may not be the best approach for people with color blindness, so alternatively other visual approaches such as pie charts, use of different shapes or percentages should also be considered to explain risk or other complex topics (e.g., side effects). It should also be born in mind that preferences regarding graphics and visuals can vary considerably between individuals.

“*It is not about wording - it is about format and layout. One way of making the information provided easier is by adding a box with bullet points at the end of each section. These bullet points would act as natural pauses between sections of the participant information sheet and would also act as a reminder for the participants of the information just read.”*

“*Information about risks is very important and it should be communicated in different ways, not only with words, but using visuals too, e.g., traffic lights, pie charts…”*

### 3.3 Beyond the provision of information: promoting respect, recognition, and wellbeing

Not only the physical materials but also the relationship with the researchers can play a key role in the consent process. Researchers are trusted and expected to have empathy and the skills to communicate the relevant information and ensure that the person is able to understand it and feels comfortable in the process. Empathy and communication skills are particularly important during face-to-face informed consent processes, as potential participants often rely on researchers to explain and provide additional information during these interactions. Having enough time to take the decision and, if appropriate, to discuss this with other people was also identified as an important issue.

“*It is necessary that the doctors put themselves in our place, sometimes they explain 50 things to you and when you leave you don't know what they have told you, empathy and clearer things are very important aspects.”*

“*Although all this information is well written, the most important thing is that they (the researchers) tell you, that they explain it well to you.”*

“*The problem is the time, you give this document to me to sign and I can talk to my children and they say, dad did you understand this? Maybe someone in the family who is a doctor can help. The problem is that there is no time. Either you sign or you are out of the study, and sometimes you sign out of desperation.”*

Easy-to-read and accessible materials can also have an important impact on the person's wellbeing and on existing misconceptions about dementia. Excessively long, technical or complicated documents may cause avoidable distress to the person, or place them in a situation of having to ask for or rely on support from others. Researchers should be able to present complex information in a lay and accessible way, rather than relying on the capacity of people to understand technical terminology and jargon.

The language used in participant-facing documents such as Informed Consent Forms should be appropriate and respectful. For example, in some consent forms whilst the participants who do not have the condition under study (the “healthy volunteers”) are referred simply as participants, the participants who have cognitive impairment are referred to as “patients.” This was perceived as an unnecessary distinction as in the context of research all groups are equally contributing to the research and are not necessarily patients. Moreover, it reflects the processes of labeling, stereotyping, and, potentially, devaluation, which are key components of stigma.

Research participation may often be about benefit to society, future generations, and one's family, rather than direct benefit. This benefit and the value of participating in research is not usually reflected in participant information sheets or informed consent forms. Recognizing and appreciating the value of the participation of people affected by dementia in research is important and fair as research would not be possible without them. It could also help to promote further participation of other people and help address some of the stigma and misconceptions linked to dementia. On the other hand, this can also be a challenge as it could affect free will and decision making.

“*Even with the disease, in a research study I am a participant and should be treated the same way as the participants without the disease.”*

“*An acknowledgment means that researchers recognize the importance of research participants as an active agent of the research itself. Whatever the findings are from the research, these are because of both the work of the researchers and of the participants. The participants' role is extremely important to help future generations with the disease.”*

“*Referring to the benefit to other people with similar conditions might make people feel bad or guilty about not consenting to it, so it could be like a subtle form of pressure.”*

### 3.4 New research approaches will affect the consent process in dementia

The field of dementia research is rapidly evolving with the emergence of new types of research and study designs. Among many other aspects, the use of Artificial Intelligence (AI) and data sharing/secondary use of data have become very relevant.

AI is increasingly being used in different aspects of health care and research. Many research projects use AI at some point and for different purposes. An important issue is how to explain the precise nature and extent of its use, including any potential risks, limitations or future consequences to participants during the consent process. This is further complicated by the fact that AI might not be directly related to their participation in the study (e.g., if data provided by the participant—e.g., blood sample or a brain scan—is used later on to develop a tool or a model using AI). Topics such as bias and possible discrimination, accountability and regulation, and the possible impact of AI-based tools on the patient-doctor relationship are all relevant to people affected by dementia. Further work is needed to develop information about AI and its impact in lay terms and understand the amount of information and detail that is appropriate for different scenarios.

Many people affected by dementia may be quite open and feel positive about the potential secondary use of their data for future research. However, the potential secondary use of data should be outlined when participants consent to join a study. This is complex information that must be understood by the participant in addition to the details of the actual study that they are about to join, and there is often, at that time, uncertainty about whether, how, with whom and for what purpose the data may be shared. This can result in relatively vague clauses in informed consent forms, as it may not be practical or possible to provide further details about the way their data will (or not) be shared in the future. Key concerns raised during our consultations related to the individual(s) or entity receiving the shared data, in particular whether the data will be shared with for-profit companies and the location of the researchers with whom the data would be shared e.g., particularly if this is outside of Europe. People affected by dementia were also concerned about data privacy, explaining that terms relating to anonymization of data (e.g., pseudonymization, “coded sample”) may not be widely understood. These terms are sometimes used inconsistently in the consent forms, thereby contributing to uncertainty and confusion, which is not conducive to promoting informed consent. On the other hand, people may be reassured by the need for ethical approval and the existence of clear regulations and standards for data protection in Europe, identifying these parameters as enablers of trust in data sharing. However, the challenge remains of how to add this additional information at the time of consent when, often, other more time-critical information tends to take precedence.

## 4 Conclusion

Over the last decade, AE has actively promoted and conducted Public Involvement (PI) activities in dementia research, involving people with dementia, carers/supporters and other members of the public (e.g., people with MCI). The work described in this article was conducted under the framework of PI using a qualitative approach. This is not qualitative research, but a systematic and rigorous methodology was nevertheless used (Gove et al., 2018).

The issue of informed consent in research has been an important topic in the work of AE, with discussions taking place as part of PI activities across several EU-funded projects. Based on this work and in one recent face-to-face meeting dedicated to this topic, we can argue that key aspects of consent relate to how participants are involved, informed and supported before, during and after their participation in research. This goes beyond the specific time where the formal process of consent takes places and encompasses issues related to comprehension but also emotions, feelings and the portrayal of dementia. It includes understanding consent in a broad context and including issues related to research awareness and access to research opportunities.

Ensuring that potential participants with cognitive problems and dementia fully understand the information provided to them is a key concern which echoes other previous work in relation to consent in dementia. This includes how the information is provided and presented to the person and a relationship of trust and respect. However, it goes beyond the mere wording and length of the text. For instance, it includes other factors that can support the person whilst promoting independence and wellbeing (e.g., tone of the document and relationship with researchers). Beyond the issue of understandability, a final key factor is linked to promoting respect, autonomy, and acknowledging the contribution and the value of participation.

Consent in dementia research is complex and it is becoming even more challenging in the context of new approaches to dementia research. Involving people affected by dementia in discussions about consent and its process can help to address these old and new challenges.

The PI work described in this article is valuable in identifying important issues about consent from the perspective of people affected by dementia and could form the basis for and contribute toward qualitative research on this topic to develop a guiding framework for informed consent in European research with people affected by dementia.

## Data Availability

The original contributions presented in the study are included in the article/supplementary material, further inquiries can be directed to the corresponding author.
